# Transient developmental imbalance of cortical interneuron subtypes presages long-term changes in behavior

**DOI:** 10.1016/j.celrep.2021.109249

**Published:** 2021-06-15

**Authors:** Lorenza Magno, Zeinab Asgarian, Valentina Pendolino, Theodora Velona, Albert Mackintosh, Flora Lee, Agata Stryjewska, Celine Zimmer, François Guillemot, Mark Farrant, Beverley Clark, Nicoletta Kessaris

**Affiliations:** 1 Wolfson Institute for Biomedical Research, University College London, Gower Street, London WC1E 6BT, UK; 2 Department of Cell and Developmental Biology, University College London, Gower Street, London WC1E 6BT, UK; 3 Department of Neuroscience, Physiology and Pharmacology, University College London, Gower Street, London WC1E 6BT, UK; 4 The Francis Crick Institute, 1 Midland Road, London NW1 1AT, UK

**Keywords:** proliferation, cortex, mouse, behavior, parvalbumin, Cux2, Pten, neurodevelopmental disorders, GABAergic interneurons

## Abstract

Cortical GABAergic interneurons are generated in large numbers in the ganglionic eminences and migrate into the cerebral cortex during embryogenesis. At early postnatal stages, during neuronal circuit maturation, autonomous and activity-dependent mechanisms operate within the cortex to adjust cell numbers by eliminating naturally occurring neuron excess. Here, we show that when cortical interneurons are generated in aberrantly high numbers—due to a defect in precursor cell proliferation during embryogenesis—extra parvalbumin interneurons persist in the postnatal mouse cortex during critical periods of cortical network maturation. Even though cell numbers are subsequently normalized, behavioral abnormalities remain in adulthood. This suggests that timely clearance of excess cortical interneurons is critical for correct functional maturation of circuits that drive adult behavior.

## Highlights

• CUX2 regulates cortical parvalbumin interneuron number generation in the MGE
• Excess parvalbumin (PV) interneuron integrates into the early postnatal cortex
• Excess PV interneuron is eliminated through PTEN-dependent mechanisms
• Behavioral abnormalities persist long after abnormal interneuron excess is cleared

## Graphical abstract

**Figure:**
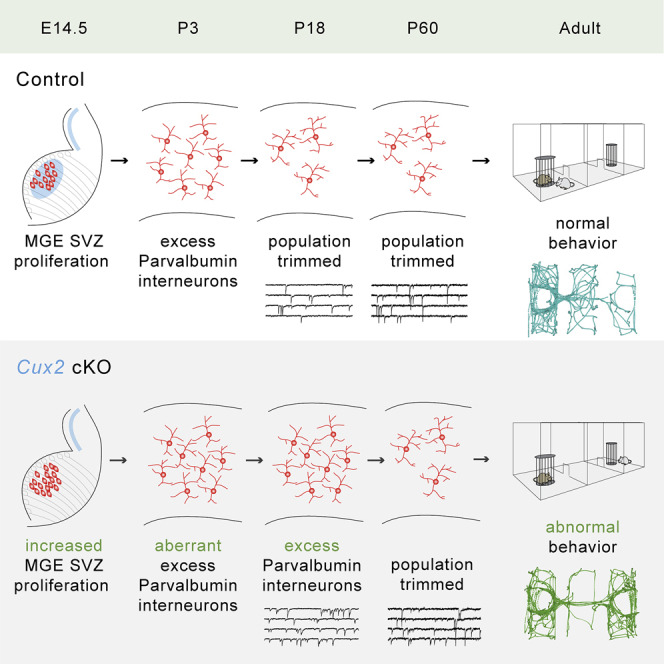

## Teaser

Cortical interneurons are generated in excess during embryogenesis, and numbers are trimmed soon after birth for optimal inhibition. Magno et al. demonstrate that aberrant parvalbumin interneuron excess persists in the postnatal cortex, spanning critical periods of cortical network maturation. Cell numbers are eventually normalized, but behavioral abnormalities remain in adulthood.

## Introduction

Cortical interneurons are normally generated in large numbers during embryogenesis, and their excess is eliminated soon after birth through cell autonomous and activity-dependent mechanisms (Southwell et al., 2012; Wong et al., 2018). The inherent capacity of the cortex to balance the excitatory/inhibitory (E/I) properties of the network by regulating cell numbers according to need is a remarkable developmental safeguarding mechanism. However, deficits in neural stem cell proliferation represent a major convergence point for human neurodevelopmental disorders (NDDs) (Ernst, 2016), suggesting a limited capacity of the cortex to protect from aberrant neuronal excess.

The embryonic ganglionic eminences (GEs) generate all the GABAergic interneurons found in the adult cortex (Bandler et al., 2017; Kessaris et al., 2014; Ma et al., 2013; Marín and Müller, 2014; Wonders and Anderson, 2006). The medial GE (MGE) is the source of two major classes of interneurons that express parvalbumin (PV) or somatostatin (SST) (Fogarty et al., 2007; Kessaris et al., 2014; Wonders and Anderson, 2006). SST interneurons are generated early, from asymmetrically dividing progenitors in the ventricular zone (VZ), whereas PV interneurons are generated later from MGE subventricular zone (SVZ) progenitors that divide symmetrically (Petros et al., 2015). The different neurogenic niche origins of these two “cardinal” interneuron classes suggest that their generation may be subject to distinct regulation.

CUX2 (cut like homeobox 2) is a homeodomain transcriptional repressor that regulates the proliferation of intermediate progenitors in the SVZ of the cortex, by promoting terminal differentiation and cell cycle exit (Cubelos et al., 2008). We detected *Cux2* expression in the SVZ of the MGE and hypothesized that it might have similar functions as in the cortex. Conditional deletion of *Cux2* in the MGE resulted in excess proliferation in the SVZ and increased numbers of PV interneurons that integrated into the early postnatal cortex. Excess PV interneurons persisted until at least postnatal day 18 (P18), after which they were eliminated through phosphatase and tensin homolog (PTEN)-dependent mechanisms. Despite this correction, mutant mice exhibited lasting behavioral deficits akin to those described in mouse models of NDDs. Our findings suggest that increased interneuron numbers, generated as a result of abnormal embryonic proliferation, constitute a transient primary defect with long-lasting behavioral consequences.

## Results

### Integration of aberrant cortical PV interneuron excess in the postnatal cortex

**Figure 1:**
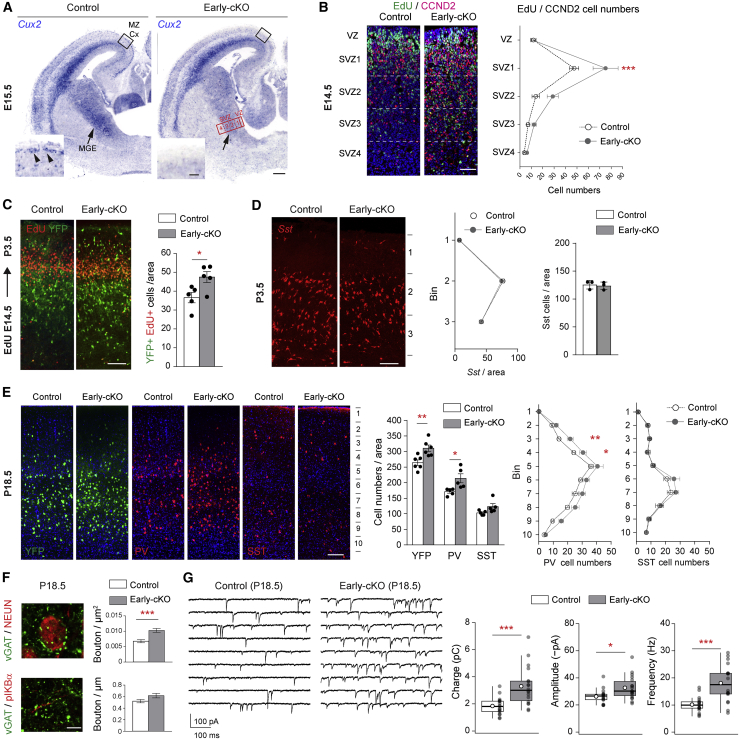
Integration of aberrant cortical parvalbumin interneuron excess in the postnatal cortex (A) Expression of *Cux2* at E15.5 and Cre recombination in *Cux2* early-cKO (Nkx2-1-Cre;Cux2^Δ/fl^) embryos. MZ-Cx, marginal zone, cortex; MGE, medial ganglionic eminence; VZ, ventricular zone; SVZ, subventricular zone. (B) Immunohistochemistry and quantification of CCND2^+ve^ cells incorporating 5-ethynyl-2′-deoxyuridine (EdU) in the MGE (red boxed area in A) at E14.5 in control (Nkx2-1-Cre;Cux2^fl/+^) and early-cKO (Nkx2-1-Cre;Cux2^fl/Δ^) embryos. n = 5 mice per group. Two-way ANOVA; genotype, p = 0.0007; zone, p < 0.0001; interaction, p = 0.01. Post hoc Bonferroni’s multiple-comparisons test. (C) Immunohistochemistry and quantification of EdU pulse-chase experiment. EdU was administered at E14.5, and EdU^+ve^YFP^+ve^ MGE-derived cells were quantified in the cortex at P3.5. Control: Nkx2-1-Cre;Cux2 ^fl/+^;R26R-YFP, early-cKO: Nkx2-1-Cre;Cux2^fl/Δ^;R26R-YFP. n = 5 pups per group. Two-tailed unpaired t test with Welch’s correction. (D) Detection of *Sst*-expressing cortical interneurons and quantification in the cortex at P3.5. n = 3 pups per group. Quantification in bins: two-way ANOVA; genotype, p = 0.6; bin, p < 0.0001; interaction, p = 0.7. Post hoc Bonferroni’s multiple-comparisons test. Total numbers: two-tailed unpaired t test. (E) Immunohistochemistry and quantification of YFP, PV, and SST in the primary somatosensory cortex barrel field at P18.5 in control (Nkx2-1-Cre;Cux2 ^fl/+^;R26R-YFP) and early-cKO (Nkx2-1-Cre;Cux2^fl/Δ^;R26R-YFP) pups. n = 5–6 mice per group. Total numbers: two-tailed unpaired t test with Welch’s correction. Quantification in bins: two-way ANOVA, PV: genotype, p < 0.0001; bin, p < 0.0001; interaction, p = 0.23. SST: genotype, p = 0.2; bin, p < 0.0001; interaction, p = 0.8. Post hoc Bonferroni’s multiple-comparisons test. (F) Immunohistochemistry and quantification of vGAT and NEUN and vGAT and pIKBα in L2/3 at P18.5. n = 132 NeuN^+ve^ cells, n = 96 AIS, 3 mice per group. Mann Whitney test, p < 0.0001. (G) Representative recordings (contiguous 1-s segments) of mIPSCs (–90 mV) from two cortical pyramidal cells (L2/3 S1 barrel field) at P18.5. Pooled data show mean mIPSC charge transfer, amplitude, and frequency (n = 17 control, 18 early-cKO cells, 4 mice per group). Box-and-whisker plots indicate median (line), 25th–75th percentiles (box), the range of data within 1.5 × interquartile range (IQR) of box (whiskers), and mean (open circles). Mann Whitney test, charge transfer, p = 0.00063; amplitude, p = 0.013; frequency, p = 0.00016. Data in (B–E) show mean ± SEM. ^∗^p < 0.05, ^∗∗^p < 0.01, and ^∗∗∗^p < 0.001. Scale bars: 250 μm (insets 100 μm) (A), 100 μm (B), 150 μm (C and D), 100 μm (E), 5 μm (F).

We detected *Cux2* expression in the SVZ of the MGE (arrows in Figure 1A) and in migrating cortical interneurons (arrowheads in insert in Figure 1A), and in order to identify the role of this transcription factor in this region, we generated a conditional mutant mouse lacking *Cux2* in the MGE at early embryonic stages (Nkx2-1-Cre;Cux2^fl/Δ^) (Figure 1A; Figure S1A). We refer to this model as the “early-conditional knockout (cKO)” in which deletion takes place in progenitors residing in the VZ of the MGE prior to the onset of neuronal migration. We confirmed the loss-of-function phenotype of the *Cux2* conditional alleles by deleting in the cortex and recapitulating the phenotype of the germline mutant mouse for which there is excess proliferation of cortical intermediate precursors and consequent increased cortical thickness (Figures S1B–S1D; Cubelos et al., 2008). In the absence of CUX2 in the MGE, we detected an increased number of proliferating CYCLIND2 (CCND2)-expressing putative PV interneuron precursors in the SVZ (Glickstein et al., 2007; Petros et al., 2015; Figure 1B; Figures S1E and S1F) and increased numbers of MGE-derived cells arriving in the cortex at P3.5 (identified by expression of yellow florescent proten (YFP) in animals carrying Nkx2-1-Cre and the Rosa26R-YFP allele) (Figure 1C). The increase in MGE cells in the cortex at P3.5 was not accompanied by an increase in *Sst*-expressing cells at this stage (Figure 1D). An excess of MGE cells was still observed in the cortex at P18.5 (YFP in Figure 1E), approximately 10 days past the peak of interneuron cell death in the cortex (Southwell et al., 2012), indicating aberrant persistence of interneuron excess. Immunolabeling for cortical interneuron subtypes showed a ∼25% increase in PV but not SST (Figure 1E) (or other non-MGE-derived interneuron populations; Figures S2A–S2D). Perisomatic inhibitory boutons onto principal cells, thought to represent maturing PV-derived boutons, were also increased (Figure 1F), as was the frequency and amplitude of miniature inhibitory postsynaptic currents (mIPSCs), resulting in a robust increase in mIPSC-mediated charge transfer (Figure 1G). To determine whether these changes were caused by a requirement for CUX2 in postmitotic MGE interneurons, which continue to express *Cux2* after exiting the MGE, we generated a “late-cKO” (Lhx6-Cre;Cux2^fl/fl^) in which *Cux2* is deleted in migrating MGE-derived interneurons (Figures S3A–S3C). All parameters examined in this mouse, including PV interneuron numbers and mIPSCs, were normal (Figures S3A–S3C). This finding indicates that, with respect to the phenotypes examined, CUX2 is dispensable in postmitotic migrating cortical interneurons. Other neurons that are generated from MGE progenitors reside in subcortical regions and include neurons of the globus pallidus, the striatum, and the amygdala (Xu et al., 2008). All populations of such neurons examined, including GABAergic (*Pv* and *Sst*) and cholinergic (*Lhx7*) neurons of the striatum, *Pv*-expressing neurons of the globus pallidus, and amygdala neurons expressing *Pv* or *Sst*, showed comparable cell densities in control and mutant brains at P18.5 (Figures S2E–S2G). This result suggests that CUX2 is either not required for the generation of normal numbers of these cells or that any excess generated in these populations is rapidly cleared. Altogether, these results indicate that CUX2 deletion in the VZ/SVZ causes increased precursor cell proliferation in the SVZ, leading to the generation of excess cortical PV interneurons that integrate into the early cortical circuits and persist beyond the peak of natural cortical interneuron cell death.

### Transient accumulation of mature PV interneurons and PTEN-dependent correction of cell numbers in the postnatal cortex

**Figure 2:**
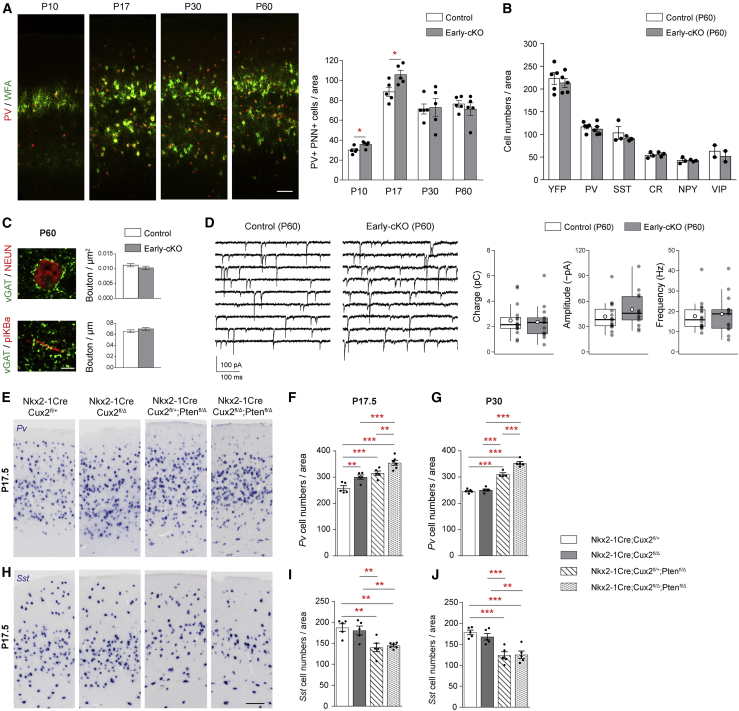
PTEN-dependent correction of cortical PV interneuron numbers in the postnatal cortex (A) Immunohistochemistry and quantification of PV^+ve^WFA^+ve^ cells in the barrel cortex of control (Nkx2-1-Cre;Cux2^fl/+^) and early-cKO (Nkx2-1-Cre;Cux2^fl/Δ^) pups and adult mice at different postnatal ages. n = 5 mice per group. Two-tailed unpaired t test with Welch’s correction,P10, p = 0.024; P17, p = 0.012. (B) Quantification of YFP, PV, and SST in the primary somatosensory cortex barrel field at P60 in control (Nkx2-1-Cre;Cux2^fl/+^;R26R-YFP) and early-cKO (Nkx2-1-Cre;Cux2^fl/Δ^;R26R-YFP) animals. n = 3–5 mice per group. Two-tailed unpaired t test with Welch’s correction. (C) Immunohistochemistry and quantification of vGAT and NEUN or vGAT and pIKBα in L2/3. n = 108 AIS, 108 NEUN^+ve^ cells, 3 mice per group. Mann Whitney test. (D) Representative recordings (contiguous 1-s segments) of mIPSCs (–90 mV) from two pyramidal cells (L2/3 S1 barrel field) at P60. Pooled data show mean mIPSC charge transfer, amplitude, and frequency (n = 16 control, 13 P60 early-cKO cells, 5 mice per group). Box-and-whisker plots as in Figure 1F. Mann Whitney test. (E–J) *Pv* mRNA expression (P17.5) (E) and quantification (P17.5 and P30) (F and G) and *Sst* mRNA expression (P17.5) (H) and quantification (P17.5 and P30) (I and J), in the primary somatosensory cortex barrel field in control (Nkx2-1-Cre;Cux2^fl/+^), *Cux2* early-cKO (Nkx2-1-Cre;Cux2^fl/Δ^), *Pten* cKO (Nkx2-1-Cre;Cux2^fl/+^;Pten^fl/Δ^), and *Cux2/Pten* double cKO mice (Nkx2-1-Cre;Cux2^fl/Δ^;Pten^fl/Δ^). One-way ANOVA, post hoc uncorrected Fisher’,s least significant difference (LSD), PV P17.5 and P30 p < 0.0001; SST P17.5 p = 0.0011; SST P30 p = 0.0033. Data in (A)–(C) and (F), (G), (I), and (J) show mean ± SEM. ^∗^p < 0.05, ^∗∗^p < 0.01, ^∗∗∗^p < 0.001. Scale bars: 100 μm (A), 5 μm (C), 200 μm (E and H).

Condensation of extracellular matrix proteoglycans into perineuronal nets (PNNs) is a measure of maturation of PV interneurons and signifies the end of critical periods of plasticity in the cortex (Pizzorusso et al., 2002). We used lectin labeling (Wisteria floribunda agglutinin [WFA]) to identify PV cells surrounded by PNNs and observed an increased number of such cells in early-cKO mice at P10 and P17 (Figure 2A). Surprisingly, by P30, this number returned to normal (Figure 2A). At P60, the number of cells expressing interneuron markers such as YFP, PV, or SST; the number of perisomatic inhibitory boutons; and all mIPSC parameters were the same as controls (Figures 2B–2D). These results indicate that there is an early accumulation of maturing PV interneurons in the cortex of the early-cKO mice, but their integration is transient, suggesting that homeostatic mechanisms may regulate interneuron numbers and their synapses in the maturing cortex.

Activity promotes the survival of young neurons through activation of the phosphatidylinositol 3-kinase (PI3K)/AKT pathway (Brunet et al., 2001; Dudek et al., 1997). PTEN antagonizes this pathway and is required for activity-dependent regulation of cortical interneuron cell death in the postnatal cortex (Wong et al., 2018). To determine whether elimination of the abnormal (as well as normal) interneuron excess requires PTEN, we examined single and compound mutants carrying conditional deletions in CUX2 and/or PTEN. At P17, mice lacking either CUX2 or PTEN had increased numbers of PV interneurons in the cortex (Figures 2E and 2F). Simultaneous conditional deletion of both CUX2 and PTEN resulted in a synergistic effect, with super-numerary PV interneurons being present in the cortex (Figures 2E and 2F). This finding is consistent with the notion that PV excess in *Cux2* early-cKO mice is caused by a mechanism independent of postnatal activity and cell survival. By P30, PV numbers were normalized in mice lacking only *Cux2*, whereas, in *Pten*-only mutants and compound *Pten/Cux2* cKO mice, they remained high (Figure 2G). Together, these data indicate that PTEN-dependent pathways continue to operate beyond the first postnatal week to normalize cell numbers and protect the network from excess GABAergic inhibition. Unlike PV interneurons, SST interneurons were decreased in the absence of PTEN at all stages examined (Figures 2H–2J), suggesting distinct roles for PTEN within the two MGE-derived cortical interneuron lineages.

### Persistent behavioral deficits in *Cux2* early-cKO mice

**Figure 3:**
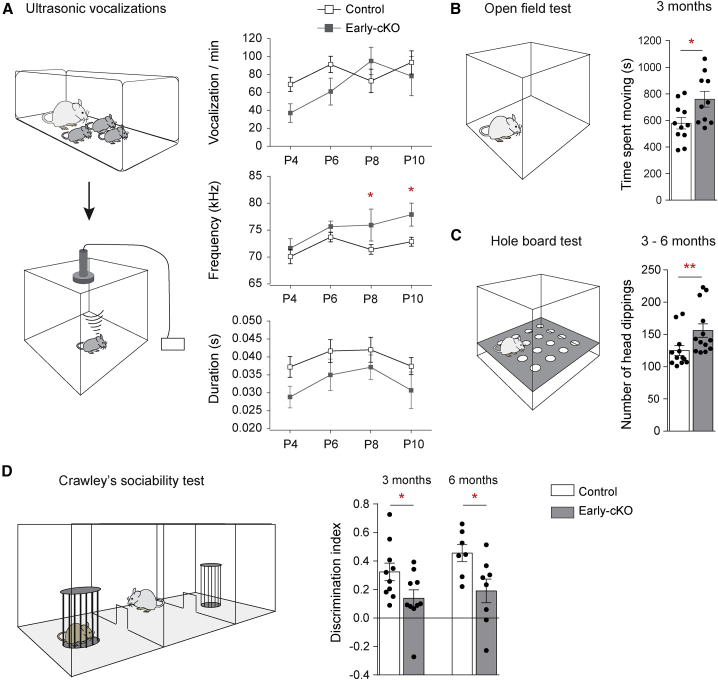
Behavioral deficits in *Cux2* early-cKO mice (A) Ultrasonic vocalizations of newborn pups when separated from the mother at P4, P6, P8, and P10. Test layout and quantification. n = 9 control, 7 early-cKO. Vocalizations: two-way ANOVA; genotype, p = 0.10; age, p < 0.0001; interaction, p = 0.97. Post hoc uncorrected Fisher’s LSD. Mean peak frequency: two-way ANOVA; genotype, p = 0.027; age, p = 0.013; interaction, p = 0.50. Post hoc uncorrected Fisher’s LSD. Duration: two-way ANOVA; genotype, p = 0.0095; age, p = 0.19; interaction, p = 0.98, Post hoc uncorrected Fisher’s LSD. (B) Open field test layout and quantification. n = 11 control, 10 early-cKO. Two-tailed unpaired t test with Welch’s correction, p = 0.025. (C) Hole board test layout and quantification. n = 12 control, 13 early-cKO. Mann Whitney test, p = 0.0052. (D) Crawley’s sociability test layout and quantification. Discrimination index [(time spent with mouse – time spent with empty cage)/(sum of the time spent with both)] at 3 and 6 months of age. 3 months: n = 10 control, 10 cKO; 6 months: n = 7 control, 8 cKO. Two-tailed unpaired t test with Welch’s correction, 3 months p = 0.043, 6 months p = 0.024. All data shown are mean ± SEM. ^∗^p < 0.05. ^∗∗^p < 0.01.

Given the significance of PV interneurons in the maturation of the cortex during critical periods of development and the hypothesis that some NDDs may represent critical period disorders (Dehorter and Del Pino, 2020; LeBlanc and Fagiolini, 2011), we assessed our mice for a range of behaviors normally associated with NDD phenotypes (Silverman et al., 2010). Where relevant, we examined both early- and late-cKO mice, in order to identify phenotypes caused by deletion of CUX2 in the SVZ, versus deletion in postmitotic MGE-derived neurons. Soon after birth, early- but not late-cKO pups showed altered communication, emitting calls of higher duration and frequency when separated from the mother (Figure 3A; Figure S3D). Adult mice showed normal general health, normal grooming (Figure S3G), lack of stereotypic or other unusual home cage behaviors (Figure S3F), and lack of anxiety-like behaviors in the open field test (Figure S3E). However, adult early- but not late-cKO mice were hyperactive in the open field test (Figure 3B; Figure S3E) and showed increased perseverative/exploratory behavior in a hole board test (Figure 3C; Figure S3H). Social behavior was assessed in the three-chambered Crawley’s sociability paradigm (Moy et al., 2004). Early-cKO mice exhibited deficits in sociability at 3 months and 6 months of age, and these defects were absent in late-cKO mice (Figure 3D; Figure S3I). Altogether, our data show persistent behavioral deficits in adult animals that have experienced transient cortical PV interneuron excess in the early postnatal cortex.

## Discussion

Our findings demonstrate that aberrant PV interneuron excess, caused by embryonic proliferation defects in the SVZ of the MGE, can be corrected by normal homeostatic mechanisms, albeit over a longer period of time, causing excess interneurons to persist through critical periods of cortical maturation. This transient integration of superfluous PV interneurons into the cortex is associated with behavioral alterations that continue long after normal interneuron numbers are restored. These findings suggest that network imbalance, caused by transient PV interneuron excess during critical periods, might alter the normal trajectory of cortical network maturation.

The regulation of cortical interneuron cell numbers is a continuous multi-step process, starting from progenitor divisions in the VZ and SVZ of the MGE, when the size of the starting population is defined. CUX2 plays a role in SVZ divisions of the MGE where, by analogy to its function in the cortical SVZ (Cubelos et al., 2008), it may regulate cell cycle exit of PV precursors. Subsequent migration and dispersal of interneurons within the cortex are subject to extrinsic and intrinsic signals during embryogenesis. Final numbers of cortical interneurons are set through maturation, network integration, and cell death, events that are coordinated by neuronal activity at early postnatal stages (Denaxa et al., 2018; Priya et al., 2018; Wong et al., 2018). Immature prospective PV interneurons initially form anatomical and functional assemblies that protect them from apoptosis before merging into the fully functional network (Duan et al., 2020; Modol et al., 2020). Our finding that excess PV interneurons form functional synapses onto pyramidal neurons and accumulate PNNs suggests that these neurons integrate into the early network; this shields them initially from cell death but does not protect them in the longer term. We have not detected extensive cell death in the cortex of juvenile mutant mice (not shown), possibly due to rapid clearance of dying cells and the transient expression of apoptotic markers (Fricker et al., 2018). In addition, the establishment of network balance is a gradual process with continuous reciprocal information flow between inhibitory and excitatory neurons that ultimately refines cell numbers to achieve an optimal E/I balance. Therefore, excess interneurons integrate and mature in our model, but their number declines gradually to normal levels through natural homeostatic mechanisms.

Despite the correction of cortical PV interneuron numbers in the juvenile cortex, behavioral abnormalities persist at adult stages in our early-cKO mouse model. The late-cKO model appears normal in regard to all phenotypes examined, narrowing down the possible root cause of behavioral defects to the embryonic SVZ of the MGE, where *Cux2* is expressed. Striatal, globus pallidus, and amygdala neuron numbers are unaffected in our early-cKO mouse, as are cortical *Sst* interneuron numbers, which is in line with their distinct niche origins (Petros et al., 2015) and molecular specification pathways (Mayer et al., 2018; Mi et al., 2018). Although we cannot exclude the possibility that other defects may occur in our early-cKO mice, our data lead us to suggest that it is the transient cortical PV interneuron excess in the cortex that is the primary cause of the behavioral phenotype. This idea is supported by the knowledge that (1) PV interneurons form the first cortical neural assemblies and a framework onto which the entire network is built (Duan et al., 2020; Modol et al., 2020), and hence, early defects in these cells are likely to result in subsequent network adjustments; (2) PV interneurons of the primary sensory areas are the first to mature (del Río et al., 1994), and hence, transient excess numbers are more likely to integrate and survive in these areas; and (3) cortical PV interneurons and their maturation timing are key factors in the timing of critical periods of cortical maturation (Takesian and Hensch, 2013). Therefore, transient abnormalities in PV cortical interneuron cell numbers—deficiency or excess—during the formative stages of the cortex are likely to leave lasting marks on the emerging network and subsequent behaviors.

The mechanism through which transient cortical PV interneuron excess may impact the cortex in the long term is unknown. It has recently been reported that in mice with a transient excess of cortical PV interneurons, caused by embryonic depletion of microglia, PV hyperinhibition and impairment of sensory information flow in the juvenile cortex are followed by long-term hypoinhibition (Thion et al., 2019). This suggests a long-term impact on the remaining PV network itself, perhaps through synaptic connectivity deficits. Alternatively, or in addition, transient PV excess may adversely impact other transient cortical circuits (Molnár et al., 2020), further disrupting the early cortical frameworks. The pivotal role of PV interneurons in the early postnatal cortex as coordinators of local cortical network development with sensory signals and gatekeepers of cortical plasticity and maturation (Di Cristo et al., 2007; Hensch, 2005) renders the cortex particularly vulnerable to variation in the number of these cells at early postnatal stages. We hypothesize that PV interneuron excess and hyperinhibition (Kirmse et al., 2015; Valeeva et al., 2016) during early postnatal development, when neurons and neural circuits are undergoing maturation (Berardi et al., 2000; Hensch, 2005), might shift the timing of critical plasticity periods in the cortex. This shift would have cascading consequences, de-synchronizing primary sensory circuit maturation, altering functional brain connectivity, and derailing the integration of sensory information and responses to sensory stimuli. Such changes would culminate in behavioral aberrations in the longer term (Takesian and Hensch, 2013). In line with this idea, recent findings show that transient enhanced cortical activity at early postnatal stages leads to long-lasting structural and functional alterations in the network and behavioral impairments in adult animals (Bitzenhofer et al., 2021). These findings highlight the importance of balanced activity during critical periods of maturation for normal cortical functions in later life.

Autism spectrum disorder (ASD) and other NDDs are disorders of higher cognitive function that are thought to be caused by underlying defects in primary sensory perception (Belmonte et al., 2004; Thye et al., 2018). Hence, normal development of primary sensory areas during the formative stages of cortical maturation is critical for higher cognitive behaviors. Our findings, and those of others, emphasize the importance of assessing cortical development from early embryonic stages through to functional networks in the adult cortex, in order to fully understand primary defects that can lead to abnormal behaviors.

Our study, as well as previous work, implicates CUX2 and CYCLIND2 in cortical PV interneuron development. Although *CCND2* has not been identified as an ASD candidate in humans to date (SFARI Gene database, https://gene.sfari.org/), mutations in *CCND2* cause megalencephaly syndrome in humans (Mirzaa et al., 2014), a phenotype often observed in ASD. *CUX2*, on the other hand, is a hotspot for *de novo* missense mutations in NDDs (Geisheker et al., 2017), including ASD (Barington et al., 2018; Chatron et al., 2018; De Rubeis et al., 2014; Geisheker et al., 2017) and has also been associated with bipolar disorder (Glaser et al., 2005; Jacobsen et al., 2001). Our model of conditional loss of CUX2 in cortical MGE interneurons does not mimic a specific human mutation in *CUX2*. However, our findings raise the possibility that defects in the SVZ of the MGE, caused by mutations in CUX2 or other regulators of MGE SVZ proliferation, may translate into transient defects with lasting behavioral deviations.

Dysfunction of cortical interneurons has been implicated in NDDs such as ASD (Hattori et al., 2017; Marín, 2012). Common among multiple mouse models of ASD are PV-specific cell number reductions and circuit defects (Gogolla et al., 2009). Reports of excess interneurons in humans are scarce, and NDDs that implicate interneuron defects have been associated with decreased rather than increased inhibition. However, our only window into early human brain development and ASD comes from recent human iPSC and organoid studies. Intriguingly, overproduction of cortical interneurons is emerging as a common finding in cultures of human cells carrying mutations in strong ASD candidate genes (Mariani et al., 2015; Paulsen et al., 2020). This leads us to propose that cortical interneuron excess, a defect that is itself transient and untraceable in later life, may underlie some forms of NDD with yet undiscovered primary etiology.

### Limitations of study

We postulate that the transient cortical PV interneuron excess observed in our *Cux2* early-cKO mice is the primary cause of behavioral abnormalities in postnatal animals. It remains possible, however, that other defects beyond the changes in cortical interneuron numbers may contribute to the observed phenotype. These defects may include those in MGE-derived cortical interneurons or elsewhere in the forebrain.

We report that excess interneurons observed in our *Cux2* early-cKO mice at early postnatal stages integrate into the cortical circuits because we find increased numbers of inhibitory synaptic puncta on pyramidal neuron cell bodies and increased inhibitory currents in pyramidal neurons. However, we have not determined the extent to which excess interneurons participate in early cortical circuitry.

Finally, we propose that there is an association between transient developmental imbalance of cortical interneuron subtypes and long-term behavioral alterations. We have not pinpointed the precise circuits that might be disrupted and which may contribute to the behavioral phenotype. The defect may be restricted to specific circuits involved in behaviors tested in this study or may arise from broader deficits in functional connectivity or network activity. Further studies are needed to identify the long-term impact on cortical circuits of transient inhibitory neuron imbalance.

## STAR★Methods

### Key resources table

**Table:** 

REAGENT or RESOURCE	SOURCE	IDENTIFIER
**Antibodies**		

Rat anti-GFP IgG2a	Nacalai Tesque	Cat # 04404-26; RRID: AB_10013361
Rabbit anti-Calretinin	Swant	Cat # 7697; RRID: AB_2721226
Mouse anti-Parvalbumin	Swant	Cat # 235; RRID: AB_10000343
Rabbit anti-SST	Peninsula Labs	Cat # T-4103; RRID: AB_518614
Rabbit anti-NPY	ImmunoStar	Cat # 22940; RRID: AB_2307354
Mouse anti-NeuN	Chemicon-Millipore	Cat # MAB377; RRID: AB_2298772
Guinea pig anti-vGAT	Synaptic Systems	Cat # 131004; RRID: AB_887873
Rabbit anti- phospho-IκBα	Cell Signaling Technology	Cat # 2859; RRID: AB_561111
Rabbit anti-CCND2	Santa-Cruz Biotechnology	Cat # sc-593; RRID: AB_2070794
Rabbit anti-Tbr2	Abcam	Cat # ab23345; RRID: AB_778267
Alexa Fluor-conjugated secondary antibodies	Invitrogen	N/A
Biotin-conjugated donkey anti-rabbit IgG	Merck Life Science UK Ltd	Cat # AP182B; RRID: AB_92587
Horseradish peroxidase-conjugated anti-DIG	Merck Life Science UK Ltd	Cat # 11207733910; RRID: AB_514500

**Chemicals, peptides, and recombinant proteins**		

Bicuculline methiodide	Tocris	Cat # 2503
Gabazine	Abcam	Cat # ab120042
Tetrodotoxin citrate	Tocris	Cat # 1069
Kynurenic acid	Sigma-Aldrich	Cat # K3375
Biotinylated Wisteria Floribunda Lectin	Vector labs	Cat # B-1355
Streptavidin-647	Life Technologies	Cat # S32357
5-ethynyl-2′-deoxyuridine	Insight Biotechnology	Cat # sc-284628A

**Critical commercial assays**		

Click-iT EdU AlexaFluor-647 Imaging Kit	Thermo Fisher Scientific	Cat # C10340
TSA Signal Amplification System	Perkin Elmer	Cat # NEL701A001KT

**Experimental models: Organisms/strains**		

Mouse: Cux2^fl/fl^	This study	N/A
Mouse: Emx1-iCre	Kessaris et al., 2006	N/A
Mouse: Nkx2-1-iCre	Kessaris et al., 2006	N/A
Mouse: Lhx6-iCre	The Jackson Laboratory	JAX: 026555; RRID: IMSR_JAX:026555
Mouse: Pten^fl/fl^	The Jackson Laboratory	JAX: 006440; RRID: IMSR_JAX:006440
Mouse: Rosa26R-YFP	The Jackson Laboratory	JAX: 006148; RRID: IMSR_JAX:006148

**Oligonucleotides**		

Cux2 WT Genotyping Forward: AGTGCTGGTAGAGATGTTGCC	This study	N/A
Cux2 WT Genotyping Reverse: TCCAATGGGAACCTTTGTCGC	This study	N/A
Cux2 fl Genotyping Forward: GCGTATTCAACAAGGGGCTG	This study	N/A
Cux2 fl Genotyping Reverse: CCTTGATGCCGTTCTTCTGCTTGT	This study	N/A
Cux2 Δ Genotyping Forward: CTGGACACATACTCCATCACC	This study	N/A
Cux2 Δ Genotyping Reverse: CCTTGATGCCGTTCTTCTGCTTGT	This study	N/A
Pten fl Genotyping Forward: CAAGCACTCTGCGAACTGAG	This study	N/A
Pten fl Genotyping Reverse: AAGTTTTTGAAGGCAAGATGC	This study	N/A
R26R-YFP WT Genotyping Forward: AAAGTCGCTCTGAGTTGTTAT	This study	N/A
R26R-YFP WT Genotyping Reverse: GGAGCGGGAGAAATGGATATG	This study	N/A
R26R-YFP KI Genotyping Forward: AAAGTCGCTCTGAGTTGTTAT	This study	N/A
R26R-YFP KI Genotyping Reverse: GGAGCGGGAGAAATGGATATG	This study	N/A
iCre Genotyping Forward: GAGGGACTACCTCCTGTACC	This study	N/A
iCre Genotyping Reverse: TGCCCAGAGTCATCCTTGGC	This study	N/A

**Recombinant DNA**		

*Vip* cDNA Clone	Source Bioscience	IMAGE: 30249277
*Pv* cDNA Clone	Source Bioscience	IMAGE: 4925213
*Sst* cDNA Clone	Source Bioscience	IMAGE: 4218815
*Cux2* cDNA Clone	Source Bioscience	IMAGE: 30532644
*Cux2* exon 23 cDNA	This study	N/A
*Lhx7* cDNA	Dr Vassilis Pachnis	N/A

**Software and algorithms**		

IGOR Pro	WaveMetrics	https://www.wavemetrics.com
TaroTools	Taro Ishikawa, Jikei University School of Medicine, Japan	https://sites.google.com/site/tarotoolsregister/
R	The R Foundation for Statistical Computing	http://www.r-project.org/
Prism	Graphpad Software, Inc	https://www.graphpad.com/scientific-software/prism/
CellProfiler	Broad Institute	https://cellprofiler.org
Volocity	Perkin Elmer	https://www.perkinelmer.com:443/lab-products-and-services/resources/whats-new-volocity-6-3.html
WinWCP/WinEDR	Strathclyde Electrophysiology Software	http://spider.science.strath.ac.uk/sipbs/software_winWCP.htm
AxoGraph	AxoGraph	https://axograph.com
Actual Track	Actual Analytics Ltd.	https://www.actualanalytics.com
Avisoft SASLab Pro	Avisoft Bioacoustics	http://www.avisoft.com
ZEISS ZEN lite	Zeiss	https://www.zeiss.com/microscopy/int/products/microscope-software/zen-lite.html
Photoshop CC & Illustrator CC	Adobe Systems Incorporated	https://www.adobe.com/
Image Composite Editor (ICE)	Microsoft	https://www.microsoft.com/en-us/research/product/computational-photography-applications/image-composite-editor/

### Resource availability

#### Lead contact

Further information and requests for resources and reagents should be directed to and will be fulfilled by the lead contact, Nicoletta Kessaris (n.kessaris@ucl.ac.uk).

#### Materials availability

Mouse lines generated in this study are available on request and are subject to MTA agreement.

#### Data and code availability

The datasets supporting the current study are available from the corresponding author on request.

### Experimental models and subject details

#### Mice

For the generation of a conditional *Cux2* allele, a targeting vector was assembled by Gene Bridges GmbH (Heidelberg). Briefly, the vector was designed to insert a loxP site at a *Sex*A1 restriction site located between exons 20 and 21 and a second loxP site in a *Stu*I site located downstream of exon 23. This was followed by a SA-STOP-IRES-Venus-pA-frt-Neo-frt cassette. Gene targeting was carried out in R1 129Sv ES cells according to standard protocols. Mice were generated by blastocyst injection. The neomycin resistance cassette used for ES cell gene targeting and selection was removed by FLP excision prior to the mice being used for experiments.

We generated a germline loss-of-function (LOF) allele for *Cux2* by crossing the conditional *Cux2* to a mouse expressing Cre in the germline. We refer to this germline-deleted allele as Cux2Δ. This was used in combination with the floxed allele in cases where fast and efficient recombination of the locus was desirable (early-cKO). In contrast, mice carrying two floxed alleles were used for the late-cKO model.

The other animals used in this study were Emx1-Cre (Kessaris et al., 2006), Nkx2-1-Cre (Kessaris et al., 2006), Lhx6-Cre (Fogarty et al., 2007) (JAX: 26555), Pten^fl/fl^ (Lesche et al., 2002) (JAX: 006440) and R26R-YFP (Srinivas et al., 2001) (JAX: 006148), all of which have been described previously. Animals were maintained on a mixed CD1/C57BL6/CBA background at the Wolfson Institute for Biomedical Research.

An initial neurophysiological screening was performed on all adult mice (3 months old at the beginning of the tests) in order to broadly assess sensory and motor function as well as general health. Male mice were used in all behavioral tests. All mice used were group housed (maximum 5 animals per cage) in a room with 12-hour light and 12-hour dark cycle and with food and water *ad libitum.* Experiments took place during the 12-hour light cycle between 09:00 to 17:00 in a room where external sounds were masked by white noise. All sessions were video-recorded for analysis purposes.

All procedures for the care and treatment of animals were in accordance with the Animals (Scientific Procedures) Act 1986.

### Method details

#### Tissue processing and immunohistochemistry

Tissue processing and immunohistochemistry were carried out as previously described (Fogarty et al., 2007). The morning of the vaginal plug was considered embryonic day (E) 0.5. The morning when a litter birth was observed, was set as postnatal day (P) 0.5.

Primary antibodies used were the following: rat anti-GFP IgG2a (1:1000 Cat # 04404-26; Nacalai Tesque, Kyoto, Japan), rabbit anti-Calretinin and mouse anti-Parvalbumin (all 1:1000 Cat # 7697, 235 Swant), rabbit anti-SST (1:200, Cat # T-4103, Peninsula Labs), rabbit anti-NPY (1:1000, RayBiotech), mouse anti-NeuN (1:1000, Cat # MAB377, Chemicon-Millipore), guinea pig anti-vGAT (1:500, Cat #131004, Synaptic Systems), rabbit anti- phospho-IκBα (1:1000 Cat # 2859, Cell Signaling Technology), rabbit anti-CCND2 (1:500, Cat # sc-593, Santa-Cruz Biotechnology), rabbit anti-Tbr2 (1:200 Cat # ab23345, Abcam). Alexa Fluor conjugated secondary antibodies were all used at 1:1000 (Invitrogen). For immunodetection of CCND2, biotin-conjugated donkey anti-rabbit IgG (1:500; Millipore) secondary antibody was applied for 1 hr at room temperature followed by Avidin/Biotinylated enzyme Complex (ABC) and Tyramide Signal Amplification (TSA) as described previously (Rubin and Kessaris, 2013). Tyramide-Cy3 (Perkin Elmer) was diluted at 1:100 and the color was developed for 3 minutes at room temperature.

*In situ* hybridization (ISH) was carried out as described previously (Fogarty et al., 2007). For ISH on fixed embryonic brains, 20 μm sections were used whereas ISH on postnatal brains was carried out on 30 μm sections. For detection of *Cux2* transcripts and Cre-mediated recombination in cKO embryos, we used a PCR-amplified template spanning exon 23 for the generation of the probe. The probes for detecting *Vip*, *Pv* and *Sst* transcripts were generated using as template IMAGE clones IMAGE: 30249277, IMAGE: 4925213, and IMAGE: 4218815, respectively (Source Bioscience).

Fluorescent *In situ* hybridization (FISH) was carried out as described above for ISH with the exception that detection was performed using a horse-radish peroxidase conjugated anti-DIG antibody followed by Tyramide Signal Amplification (TSA) detection. Tyramide-Cy3 (Perkin Elmer) was diluted at 1:100 and the color was developed for 3-4 hours at room temperature. The protocol has been described in Harris et al. (2018).

For detection of perineuronal nets Biotinylated Wisteria Floribunda Lectin (1:1000, Cat # B-1355, Vector labs) was applied on sections for 1 hour, followed by Streptavidin-647 (Cat #S32357, Life Technologies). Immunohistochemistry for PV and YFP on the same slides was carried out as described previously.

#### EdU administration and detection

5-ethynyl-2′-deoxyuridine (EdU, Molecular Probes) was dissolved in sterile PBS at 2.5 mg/ml. Pregnant females were administered an intraperitoneal injection of EdU (10 mg/Kg bodyweight). Dams were sacrificed 30 minutes following injection or were allowed to give birth and pups were sacrificed at P3.5 for pulse-chase experiments. EdU detection was carried out after CCND2 or YFP immunohistochemistry using the Click-iT EdU AlexaFluor-647 Imaging Kit (Molecular Probes) according to manufacturer’s instructions and as described previously (Magno et al., 2012).

#### Imaging and quantification

Unless otherwise stated, images were captured using a Hamamatsu C4742-95 camera attached to a Zeiss Axioplan fluorescence microscope and associated Digital Pixel software. Image composites were assembled using Microsoft ICE software (Microsoft Corp., Redmond, WA) and processed with Adobe Photoshop CC (Adobe Systems Inc., San Jose, CA) for general contrast and brightness enhancements. Figures were generated using Adobe Illustrator CC (Adobe Systems Inc., San Jose, CA). Images of RNA ISH were taken using a ZEISS Axio Scan.Z1 and processed using ZEISS ZEN lite software.

#### MGE proliferation

for quantification of proliferation markers in the embryonic MGE, 15 μm cryosections were stained for the relevant markers and four sections of MGE at defined anterior-posterior levels were identified for each mouse. Composites of single confocal optical frames of the MGEs were taken using a Leica CTR6500 confocal microscope and counts were performed in an area spanning 200 μm x 600 μm of the MGE proliferative zones, as indicated in the text.

#### EdU pulse-chase experiments and quantification at P3.5

Briefly, 30 μm sections were stained for EdU and YFP or Sst ISH, as described, and the relevant areas were imaged. Counts were performed in 550 μm width x 30 μm depth areas spanning the entire dorso-ventral extent of the cortex.

#### Neuron numbers: Cortex

cortical interneuron numbers were counted on composite images of 400 μm width x 30 μm depth areas spanning the entire dorso-ventral extent of the cortex as previously described (Magno et al., 2012). Where relevant, the cortex was divided into 10 equal bins for quantification purposes. Cortical layer 1 corresponds to bin 1, layers 2/3 largely span bins 2-5, layer 4 corresponds to bin 6 and layers 5/6 largely span bins 7-10. Counts in Figure S3B were generated using Cell profiler software. Cortical interneuron numbers on images of RNA ISH were counted on composite images of 1000 μm width x 30 μm depth areas spanning the entire dorso-ventral extent of the cortex. Other areas: neuron numbers in other areas were quantified on images generated in ZEN lite software and are presented as cell densities.

#### Synaptic puncta

Quantification of synaptic markers was carried out on confocal images acquired on a Perkin- Elmer spinning disc microscope (Leica SPE2). Serial square planes of 0.114 μm x 0.114 μm were obtained for each color channel using 63x magnification with a z-step of 0.42 μm. One section per animal was used to obtain four confocal stacks, each of which contained more than ten nuclei or axon initial segments. We obtained 12 planes per stack for quantification of synapses onto AIS and 26 planes per stack for the perisomatic synapses. Images were analyzed using Perkin Elmer Volocity Software.

Perineuronal net counts were performed on composite images of 400 μm width x 30 μm depth areas spanning the entire dorso-ventral extent of the cortex.

#### Brain slice preparation

To prepare acute brain slices from ‘early-cKO’ or ‘late-cKO’ and WT littermate mice, male and female mice (P18–19) were deeply anesthetized with isoflurane and decapitated. The brain was removed and submerged in ice-cold slicing solution containing 125 mM NaCl, 2.5 mM KCl, 2.5 mM MgCl_2_, 1.25 mM NaH_2_PO_4_, 0.5 mM CaCl_2_, 25 mM d-glucose, and 26 mM NaHCO_3_, saturated with 95% O_2_ and 5% CO_2_, pH 7.4. In some cases, CaCl_2_ was reduced to 0.5 mM, and MgCl_2_ increased to 4 mM. Coronal slices (250 μm thick) containing somatosensory ‘barrel’ cortex were prepared using a vibratome (Leica VT12000S or Campden 7000smz). Before recording, slices were allowed to recover in recording solution (as above, but with 2 mM CaCl_2_ and 1 mM MgCl_2_) for 30 minutes at 32–34°C and thereafter at room temperature for 30 minutes. To prepare slices from older (P60) ‘early cKO’ and WT littermate mice, the same methods were used but the slicing solution contained 120 mM K-gluconate, 15 mM KCl, 20 mM HEPES, 25 mM d-glucose, 4 mM Na-pyruvate, 0.05 mM EGTA and 10 mM Na-ascorbate saturated with 100% O_2_, pH adjusted to 7.4 with KOH. Individual slices were transferred to a submerged chamber on the stage of an upright microscope (Scientifica SliceScope or Olympus BX51WI) and perfused with recording solution at 2 ml/min. Pyramidal cells of the S1 barrel field were visualized using 40x or 60x water immersion objectives.

#### Whole-cell voltage-clamp recordings

Recordings were made using a Multiclamp 700B patch-clamp amplifier (Molecular Devices). Data were filtered at 2 or 4 kHz and digitized at 20 or 50 kHz via an ITC-18 AD board using AxoGraph or Strathclyde Electrophysiology software. Patch electrodes were pulled (Narishige PC-10) from borosilicate glass giving resistances of 3.5–6 MΩ when filled with internal solution containing 130 mM CsCl, 10 mM EGTA(Cs), 10 mM HEPES, 10 mM NaCl, 4 mM MgATP, 0.3 mM Na_2_GTP, adjusted to pH 7.3 with CsOH. Series resistance and input capacitance were read directly from the amplifier settings used to minimize the current responses to 5 mV hyperpolarizing voltage steps. Series resistance was typically compensated by 50%–80% and data were discarded if the series resistance varied by > 20%. Miniature inhibitory postsynaptic currents (mIPSCs) were recorded from the soma of layer 2-3 pyramidal cells at a command potential of –90 mV to maximize the signal-to-noise ratio. TTX (0.5 μM, Tocris) and kynurenic acid (1–3 mM, Sigma-Aldrich) were added to the external solution to block action potentials and glutamatergic synaptic currents, respectively. Bicuculline methiodide (20 μM, Tocris) or gabazine (20 μM, Abcam) were applied at the end of experiments to confirm that the recorded events were GABA_A_R-mediated. For analysis, data were digitally filtered at 2 kHz. mIPSC-mediated charge transfer was calculated using an automated procedure (custom-written in IGOR Pro 6; WaveMetrics) that avoided subjective decisions regarding detection or selection of individual synaptic currents. The record was split into 1 s segments and for each segment an all-point amplitude histogram was generated and fit with a single-sided Gaussian to the most-positive current values. The position of the peak of the histogram was taken as the baseline current for that segment and subtracted from the record. The integral of the subtracted current provided the charge carried by the synaptic events. The total charge was divided by the recording period analyzed (20–130 s) to give a measure of phasic charge transfer per second. To determine the amplitude of individual mIPSCs and their average frequency, events were detected using an amplitude threshold algorithm (TaroTools, custom-written procedure in IGOR Pro 6), where the threshold for detection was set at ∼3 times the SD of the baseline noise (typically 12–18 pA). All selections were inspected and missed events or errors corrected manually.

#### Behavioral analysis

Behavioral phenotyping was conducted in the following sequences for the following cohorts.

#### Early mutation (early-cKO)

Cohort 1: open field, marble burying, Crawley’s three-chamber test.
Cohort 2: open field, Crawley’s three-chamber test, marble burying, hole board.
Cohort 3: open field, Crawley’s three-chamber test, marble burying, hole board
Cohort 4: pup ultrasonic vocalization test, open field, hole board, Crawley’s three-chamber test.

#### Late mutation (late-cKO)

Cohort 1: pup ultrasonic vocalization test, open field, Crawley’s three-chamber test, hole board
Cohort 2: pup ultrasonic vocalization test, open field, Crawley’s three-chamber test, hole board.

#### Open field

The open field task was carried out in an acrylic 30x30x40 cm square transparent box during a 30 min period. Actual Track software (Actual Analytics Ltd., Edinburgh, UK) was used to track the mice’s movements during each session. The average speed was calculated as total distance traveled over the time spent moving. The total time spent grooming was also scored during the 30-minute test period in the open field arena by a trained observer with a stopwatch.

#### Crawley’s three-chamber test

Sociability and social memory were assessed as previously described (Nadler et al., 2004). Each mouse was placed in the apparatus for two 10 min sessions, with the first session testing social interaction, and the second one assessing social memory (preference for social novelty). The interaction was scored manually and a discrimination index was calculated as difference in the time spent interacting with the stranger mouse and the empty cage over the sum of the time spent with both. The preference for social novelty test was included as a control to confirm olfactory abilities for detection and discrimination of social odors.

#### Hole board

The task was carried out in the open field area with an elevated board floor containing 16 symmetrical holes of 2 cm diameter. A wire mesh was placed underneath the board to prevented mice going under the board. The mice were allowed to explore the area for 15 minutes. The number of head-dippings in the holes was counted as a measurement of repetitive behavior (Moy et al., 2008).

#### Marble burying test

The test was performed in a cage containing clean bedding (5 cm depth) and 12 glass marbles evenly spaced on the surface approximately 4 cm apart. Mice were placed in the cage for 15 minutes, after which the number of marbles buried by at least 2/3 of their depth were scored.

#### Ultrasonic vocalizations

Each pup was separated from the mother, placed into an empty plastic container (diameter 20 cm), located inside a sound-attenuating Styrofoam box, and assessed for USVs during a three-minute test. Ultrasonic calls were recorded in a sound-attenuating chamber by an Ultrasound Microphone (Avisoft UltraSoundGate condenser microphone capsule CM16, Avisoft Bioacoustics, Berlin, Germany) sensitive to frequencies of 10-180 kHz. The microphone was placed over the Styrofoam sound-attenuating chamber, about 20 cm above the plastic container. The temperature of the room was maintained at 22 ± 1°C. Vocalizations were recorded using Avisoft Recorder v3.2 (Avisoft Bioacoustics, Berlin, Germany) connected to a computer system (Dell Optiplex GX270). For acoustical analysis, recordings were transferred to Avisoft SASLab Pro (Version 4.40) and a fast Fourier transformation (FFT) was conducted. Spectrograms were generated with an FFT-length of 256 points and a time window overlap of 50% (100% Frame, Hamming window). The spectrogram was produced at a frequency resolution of 977 Hz and a time resolution of 0.512 ms. A lower cut-off frequency of 15 kHz was used to reduce background noise outside the relevant frequency band to 0 dB. The total number of calls and their duration were analyzed for each testing day. Additional qualitative and quantitative analyses included sound frequencies, measured in terms of peak frequencies (frequencies with the highest sound pressure), and peak amplitude at the peak frequency (maximum of the spectrum).

### Quantification and statistical analysis

Cell counts and mIPSC analyses were performed by investigators blind to the genotypes. For behavioral experiments the investigators handling the mice and analyzing the video-recordings were also blind to the genotypes. Statistical analysis was carried out using Prism 9 for Windows (GraphPad Software, La Jolla, CA) or R (v.3.3.2; the R Foundation for Statistical Computing; http://www.r-project.org/) and R Studio (v.1.0; RStudio). All data were tested for normality using a Kolmogorov-Smirnov test and subsequently analyzed using an appropriate statistical test: unpaired t test with Welch’s correction, one-way and two-way ANOVA with post hoc uncorrected Fisher’s Least Significant Difference (LSD) test or Bonferroni’s multiple comparisons test, for normally distributed data; and the nonparametric Mann Whitney test for non-normally distributed data, unless specified otherwise. All t tests were two-tailed with an alpha of 0.05.

## Supplemental information

Document S1. Figures S1–S3

Document S2. Article plus supplemental information
