# Supplementary material for: Transient developmental imbalance of cortical interneuron subtypes presages long-term changes in behavior
Source: Cell Rep. 2021 Jun 15;35(11):109249. doi: 10.1016/j.celrep.2021.109249 (PMC8220254; doi:10.1016/j.celrep.2021.109249)
Supplement: Document S1. Figures S1–S3 [file mmc1.pdf]

Cell Reports, Volume *35*

Supplemental information

Transient developmental imbalance of cortical interneuron subtypes presages long-term changes in behavior

Lorenza Magno, Zeinab Asgarian, Valentina Pendolino, Theodora Velona, Albert Mackintosh, Flora Lee, Agata Stryjewska, Celine Zimmer, François Guillemot, Mark Farrant, Beverley Clark, and Nicoletta Kessaris

Supplemental Figures

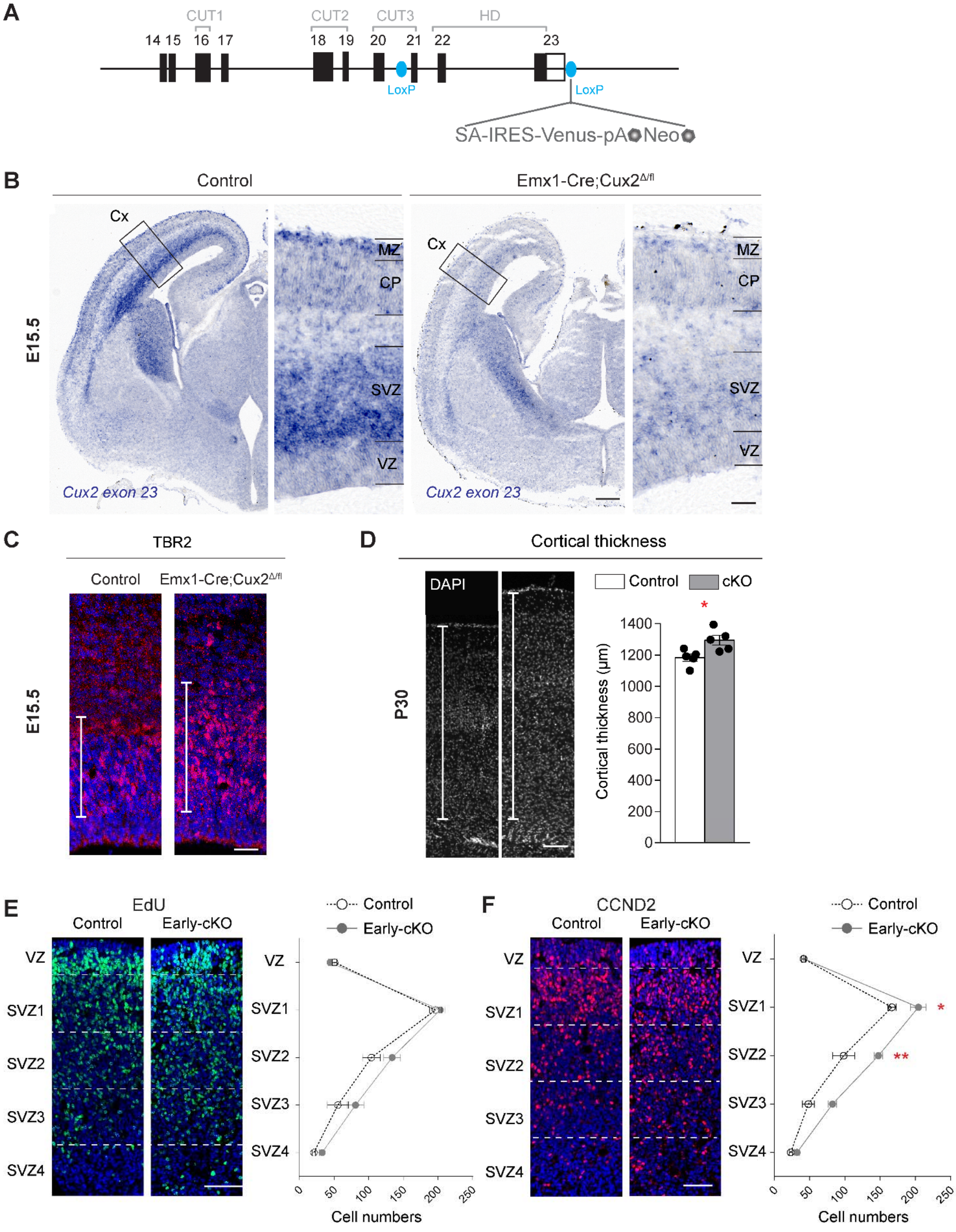

Supplemental Figure 1 (related to Figure 1)

(A – D) Generation and validation of a new Cre conditional mouse *Cux2* allele

(**A**) Schematic showing the structure of the modified *Cux2* locus: the position of loxP sites and the cassette inserted are shown. A splice acceptor (SA)-IRES-Venus cassette had been inserted downstream of the loxP site. The neomycin resistance cassette had been flanked by frt sites and had been removed prior to using the conditional *Cux2* mouse.

(**B**) Detection of *Cux2* expression in the embryonic telencephalon at E15.5 using exon 23 as a probe. Transcripts are observed in the SVZ of the cortex (see inset) and scattered cells in the MZ and the cortex. In the presence of Emx1-Cre, deletion of *Cux2* is observed in the SVZ of the cortex. Remaining *Cux2*$^{+ve}$ cells likely represent migrating subcortical-derived cortical interneurons.

(**C**) Immunohistochemistry for TBR2 at E15.5 in control and Emx1-Cre cKO embryos shows the presence of increased numbers of intermediate precursors in the SVZ of the cortex in the cKO. This recapitulates previous findings in the cortex of *Cux2* null mice and validates our conditional allele as a loss-of-function allele.

(**D**) DAPI-stained cortical images from control and Emx1-Cre;Cux2$^{\Delta/fl}$ cKO mice at P30. Increased cortical thickness in Emx1-Cre;Cux2$^{\Delta/fl}$ cKO mice, in line with findings in germline *Cux2* mutant mice. Data show Mean ± SEM, n = 5 mice per group. Two-tailed unpaired *t* test with Welch's correction, *$p < 0.05$.

CUT, cut homology domains; HD, homeodomain; SA, splice acceptor; pA, poly adenylation cassette; Neo, neomycin resistance cassette; MZ, marginal zone; CP, cortical plate; SVZ, subventricular zone; VZ, ventricular zone.

Scale bars: (B) 250 μm, (inset 50 μm), (C) 50 μm, (D) 100 μm.

(E – F) Proliferation of SVZ progenitors in the MGE in the absence of *Cux2*

(**E**) Detection and quantification of S-phase cells incorporating EdU at E14.5. Two-way ANOVA, Genotype $p = 0.09$, Zones $p < 0.0001$, Interaction $p = 0.6$. Post-hoc Bonferroni's multiple comparisons test.

(**F**) Immunohistochemistry and quantification of CCND2^{+ve} cells in control and MGE cKO embryos show increased numbers of these cells in SVZ1 and SVZ2 in early-cKO embryos. Two-way ANOVA, Genotype $p = 0.0002$, Zones $p < 0.0001$, Interaction $p = 0.08$. Post-hoc Bonferroni's multiple comparisons test.

Data show Mean ± SEM, n = 5 embryos per group. * $p < 0.05$, ** $p < 0.01$.
Scale bar: 100 μm.

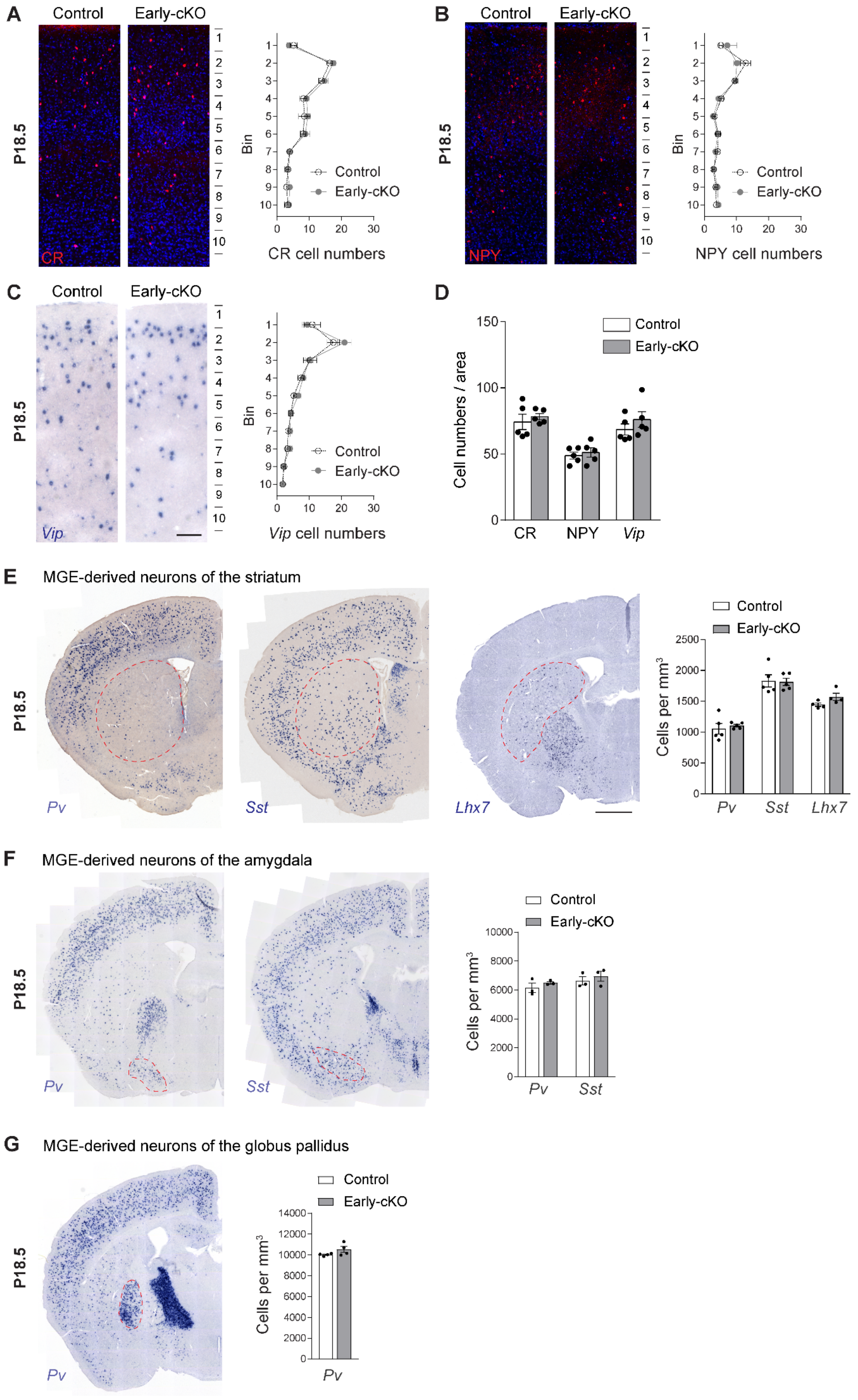
A
Control
Early-cKO
P18.5
CR
Bin
CR cell numbers
Control
Early-cKO
B
NPY
NPY cell numbers
C
Vip
Vip cell numbers
D
Cell numbers / area
CR
NPY
Vip
E
MGE-derived neurons of the striatum
Pv
Sst
Lhx7
Cells per mm3
F
MGE-derived neurons of the amygdala
G
MGE-derived neurons of the globus pallidus

Supplemental Figure 2 (related to Figure 1)

(A – D) Normal numbers of CGE-derived interneurons in the postnatal cortex at P18.5 in the absence of *Cux2* in the MGE

Immunohistochemistry and quantification of CR (**A**), NPY (**B**) and *in situ* hybridization detecting *Vip* (**C**) in the primary somatosensory cortex barrel field. n = 5 mice per group. Two-way ANOVA, CR: Genotype $p = 0.2$, Bin $p < 0.0001$, interaction 0.8. NPY: Genotype $p = 0.7$, Bin $p < 0.0001$, interaction 0.6. *Vip*: Genotype $p = 0.26$, Bin $p < 0.0001$, interaction 0.4. Post-hoc Bonferroni's multiple comparisons test.

(**D**) Total numbers of interneuron subtypes in the postnatal cortex in control and *Cux2* MGE early-cKO pups at P18.5. Two-tailed unpaired *t* test with Welch's correction.

All Data show Mean ± SEM.
Scale bar: 100 μm.

(E – G) Normal numbers of MGE-derived neurons outside the cortex at P18.5 in the absence of *Cux2* in the MGE

(**E**) *In situ* hybridization detecting MGE-derived neurons of the striatum expressing *Pv*, *Sst* or *Lhx7* and quantification of cell densities in the striatal areas indicated. n = 4 - 5 mice per group.

(**F**) *In situ* hybridization detecting MGE-derived neurons of the amygdala expressing *Pv* or *Sst* and quantification of cell densities. n = 3 mice per group.

(**G**) *In situ* hybridization detecting MGE-derived neurons of the globus pallidus expressing *Pv* and quantification of cell densities. n = 4 mice per group

Two-tailed unpaired *t* tests with Welch's correction.
All Data show Mean ± SEM.
Scale bar: 500 μm.

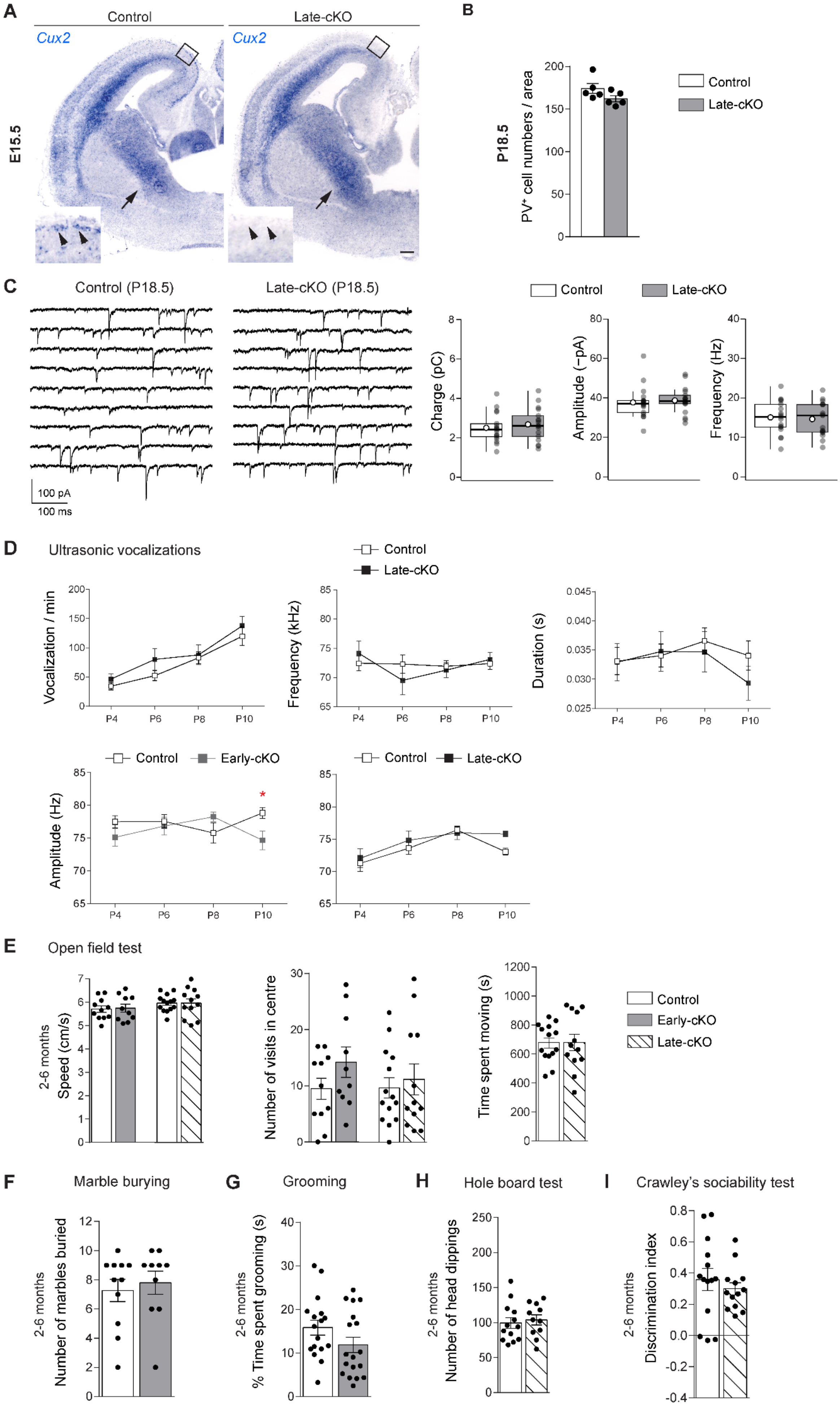

A
Control
Late-cKO
Cux2
Cux2
E15.5
B
P18.5
PV⁺ cell numbers / area
Control
Late-cKO
C
Control (P18.5)
Late-cKO (P18.5)
100 pA
100 ms
Control
Late-cKO
Charge (pC)
Amplitude (−pA)
Frequency (Hz)
D
Ultrasonic vocalizations
Control
Late-cKO
Vocalization / min
Frequency (kHz)
Duration (s)
P4
P6
P8
P10
Control
Early-cKO
Control
Late-cKO
Amplitude (Hz)
E
Open field test
2-6 months
Speed (cm/s)
Number of visits in centre
Time spent moving (s)
Control
Early-cKO
Late-cKO
F
Marble burying
G
Grooming
H
Hole board test
I
Crawley's sociability test
2-6 months
Number of marbles buried
2-6 months
% Time spent grooming (s)
2-6 months
Number of head dippings
2-6 months
Discrimination index

Supplemental Figure 3 (related to Figures 1 and 3)

(A – C) Normal numbers of cortical PV interneurons at P18.5 upon late deletion of *Cux2* in migrating MGE-derived interneurons, related to Figure 1.

(**A**) Expression of *Cux2* in the telencephalon at E15.5 and deletion in migrating MGE lineage cells in Lhx6-Cre;Cux2$^{fl/fl}$ embryos. Intact expression in the SVZ of the GE (arrows) but loss of transcripts in migrating cortical interneurons (arrowheads in insets) in Lhx6-Cre-expressing embryos. Scale bar: 250 μm

(**B**) Quantification of PV cortical interneurons in the postnatal cortex in control and late-cKO pups at P18.5. Data show Mean ± SEM. Two-tailed unpaired *t* test with Welch's correction.

(**C**) Representative recordings (contiguous 1-s segments) of mIPSCs (–90 mV) in pyramidal cells from a P18 control mouse (left) and a P18 late-cKO mouse (right) (L2/3 S1 barrel field). Below, pooled data showing no change in mean mIPSC charge transfer (n = 17 control and 17 late-cKO cells, 4 mice in each group; Mann Whitney test, p = 0.53), amplitude (p = 0.72), and frequency (p = 0.77). Box-and-whisker plots as in Figure 1.

(D – I) Behavioral analysis of control and *Cux2* early- and late-cKO mice, related to Figure 3.

(**D**) Ultrasonic vocalizations of newborn pups when separated from the mother at different postnatal ages (P4, P6, P8 and P10). Comparable call rates (vocalizations) are observed between late- *Cux2* cKO pups and controls (Late-cKO: Two-way ANOVA, Genotype p = 0.28, Age p = 0.027, interaction p = 0.088, post-hoc uncorrected Fisher's LSD). Normal mean peak frequency in late-cKO pups compared to controls (Late-cKO: Two-way ANOVA, Genotype p = 0.85, Age p = 0.29, interaction p = 0.40, post-hoc uncorrected Fisher's LSD. Normal duration of calls by late-cKO pups compared to controls (Late-cKO: Two-way ANOVA, Genotype p = 0.27, Age p = 0.49, interaction p = 0.78, post-hoc uncorrected Fisher's LSD. Late-cKO pups: n = 22 control, 11 cKO. Comparable amplitude of calls made by early- *Cux2* cKO pups and controls and between late- *Cux2* cKO pups and controls at different postnatal ages (P4, P6, P8 and P10). Early-cKO: Two-way ANOVA, Genotype p = 0.13, Age p = 0.86, interaction p = 0.058, post-hoc uncorrected Fisher's LSD. Late-cKO, Two-way ANOVA, Genotype p = 0.23, Age p = 0.0004, interaction p = 0.60, Post-hoc uncorrected Fisher's LSD. Early-cKO pups: n = 9 control, 7 cKO. Late-cKO pups: n = 20 control, 12 cKO. * < 0.05.

(**E**) Open filed test. Running speed and numbers of visits in center were comparable between control and early-cKO mice and between control and late-cKO mice during the 30-minute test period. Running speed: early-cKO: Two-tailed unpaired *t* test with Welch's correction, p = 0.85; late-cKO: Two-tailed unpaired *t* test with Welch's correction p = 0.98. Number of visits: early-cKO: Two-tailed unpaired *t* test with Welch's correction, p = 0.17; late-cKO: Mann Whitney test p = 0.99. Data show Mean ± SEM. Late-cKO mice spend normal time moving compared to their respective controls. Data show Mean ± SEM. Two-tailed unpaired *t* test with Welch's correction. Early-cKO: n = 11 control, 10 cKO; Late-cKO: n = 14 control, 12 cKO.

(**F**) Marble burying test. Early-cKO mice and controls buried comparable numbers of marbles during the 15-minute test period. Data show Mean ± SEM. n = 11 control, 10 cKO. Mann Whitney test.

(**G**) Grooming. Early-cKO mice and controls spent comparable times grooming in their home cage during the test period. Data show Mean ± SEM, n = 17 control, 18 cKO. Mann Whitney test.

(**H**) Hole board test. Normal numbers of head-dippings by late-cKO mice compared to controls. Data show Mean ± SEM. n = 13 control, 11 cKO. Two-tailed unpaired *t* test with Welch's correction.

(**I**) Crawley's sociability test. Normal discrimination index [(time spent with mouse – time spent with empty cage) / (sum of the time spent with both)] of late-cKO mice compared to controls at 3 months of age. Data show Mean ± SEM. n = 14 control, 13 cKO. Two-tailed unpaired *t* test with Welch's correction.
